# The COVID-19 pandemic in children and young people during 2020-2021: Learning about clinical presentation, patterns of spread, viral load, diagnosis and treatment

**DOI:** 10.7189/jogh.11.01010

**Published:** 2021-12-25

**Authors:** Igor Rudan, Davies Adeloye, Srinivasa Vittal Katikireddi, Josie Murray, Colin Simpson, Syed Ahmar Shah, Chris Robertson, Aziz Sheikh, EAVE II collaboration

**Affiliations:** 1 Centre for Global Health, Usher Institute, The University of Edinburgh, Edinburgh, UK; 2 MRC/CSO Social & Public Health Sciences Unit, Glasgow, UK; 3 COVID-19 Surveillance Lead, Public Health Scotland, Fife, UK; 4 School of Health, Wellington Faculty of Health, Victoria University of Wellington, New Zealand; 5 Usher Institute, The University of Edinburgh, Edinburgh, UK; 6 Department of Mathematics and Statistics, University of Strathclyde, Glasgow, UK and Public Health Scotland, Glasgow, UK

The initial research questions posed by the scientists faced with SARS-CoV-2 outbreaks were focused on documenting the clinical presentation and the characteristics of the spread of the SARS-CoV-2 virus among CYP [1-3], and the optimal ways to diagnose CYP and treat those in need [1,4]. It quickly became apparent that younger age groups have a considerably milder clinical presentation, but also that they can very rarely develop a considerably more serious Multisystem Inflammatory Syndrome in Children (MIS-C) [1]. A special interest arose in how best to protect those particularly vulnerable. In this Editorial, we discuss the development of scientific evidence related to those research questions in children and young people during the first two years of the COVID-19 pandemic, based on the information available until December 1st, 2021.

## EARLY RESEARCH ON THE SYMPTOMS AND SPREADING OF THE SARS-COV-2 AMONG CHILDREN AND YOUNG PEOPLE

Zimmermann and Curtis provided one of the earliest overviews of the epidemiology, clinical features, diagnosis and treatment in children [5]. Preliminary insights suggested that children were just as likely as adults to become infected with SARS-CoV-2, but they showed symptoms less frequently, particularly severe ones. Critically, the importance of younger age groups in spreading the virus in the population remained very uncertain [5]. Children also seemed to have gastrointestinal symptoms more often. Experts agreed that the high proportions of asymptomatic or mildly symptomatic infections in children deserved careful attention, as they could be spreading the virus more readily [6,7].

Analysis from the Robert Koch Institute in Germany early in the pandemic, in March and April 2020, demonstrated a relative increase over time in the prevalence of infections among 15-34 age group – particularly 20-24 age group – compared with 35-49 and 10-14 age groups following the introduction of physical distancing measures [8]. Understanding why the same virus causes such different symptoms across the age spectrum presented a unique opportunity to learn about disease-modifying host factors by studying young populations [9-12].

Further information continued to arrive from all over the world. Media reported on twelve critically or mortally ill children before April 6, 2020 [13]. There was concern in early Chinese studies about vertical mother-to-child transmission, with six early-onset (<7 days) and 3 late-onset neonatal SARS-CoV-2 infections found in the early literature [13]. The new virus was detected in nasopharyngeal swabs of all hospitalized children, in stool samples in 89%, and oropharyngeal swabs in 33%, but not in sera or urine samples [14]. The median duration of viral shedding was 13 days for the nasopharynx, 4 days for the oropharynx, and 43 days for stools [14]. A study of asymptomatic caretaker-child pairs in New York in May and June 2020, identified the rate of asymptomatic COVID-19 of 2.5% in hospitalized children and 7.5% in caretakers [15]. In Pakistan, recovery from COVID-19 in children has been reported as “largely universal”, with less than 2% with severe disease requiring intensive care [16].

Then, larger studies began to appear. The Paediatric Tuberculosis Network European Trials Group (ptbnet) conducted a multinational, multicentre study in 82 health care institutions from 25 European countries. They studied all minors with confirmed SARS-CoV-2 infection in April 2020, during the first wave of the pandemic in Europe, and found 582 cases. The median age was 5.0 years (IQR 0.5-12.0), the sex ratio of 1.15 males per female, 25% had pre-existing medical conditions, 62% were hospitalized, 8% required ICU admission, and 4% mechanical ventilation. Risk factors for requiring ICU admission were being younger than 1 month (OR = 5.1), male sex (OR = 2.1), pre-existing medical conditions (OR = 3.3), and presence of lower respiratory tract infection (OR = 10.5). Eventually, four children died, suggesting the case-fatality rate of 0.69% [17].

Across the Atlantic, in the United States, the report by COVID-19-Associated Hospitalization Surveillance Network (COVID-NET) was based on laboratory-confirmed COVID-19-associated hospitalizations in 14 states and covered a period between March and July 2020. They reported on 576 pediatric COVID-19, quoting the cumulative COVID-19-associated hospitalization rate among minors of 8.0 per 100 000 population, which was about 20 times less than in adults. The highest rate was observed among children <2 years (24.8/100 000). The authors noted that Hispanic and black children had higher rates (16.4 and 10.5 per 100 000, respectively) than white children (2.1). A third of hospitalized children were admitted to an ICU, about 6% required mechanical ventilation, and one patient died [18]. In the United States, minors represented 22% of the population but less than 2% of confirmed SARS-CoV-2 cases as of April 2020, with a period seroprevalence of ≈ 1% and most seropositive children not suspected of having had COVID-19 [19].

Further research consolidated the estimate that minors comprise 1%-2% of the diagnosed cases, with a median age of 3.3-11.0 years and with a male-to-female ratio of 1.15-1.55. The most common symptoms in children were upper respiratory symptoms (26%-54%), cough (44%-54%), fever (32%-65%), and gastrointestinal symptoms (15%-30%), while between 4%-54% were asymptomatic. Case-fatality rates could be up to 0.7% [20-26]. In four New York hospitals, 28% of patients required critical care, and among them 35% required respiratory support, with 9% needing mechanical ventilation, but all patients survived.

As case series of studied children grew larger, the preliminary report of pediatric COVID-19 from the US Centers for Disease Control (CDC) based on 2572 cases between February and April 2020, showed that 20% received inpatient treatment and 2% required admission to the intensive care unit – in line with early reports from other countries [26]. Larger numbers allowed for some differentiation within the minor age groups. An interesting observation was that the infected children were less likely to have asthma, that children aged 6-13 years were often asymptomatic (39%) and had respiratory symptoms less often (29% - in comparison to 48% in younger children and 60% in adolescents). Furthermore, in comparison to children aged 6-13 years, adolescents were more frequently diagnosed with influenza-like symptoms (61% vs 39%), gastrointestinal symptoms (27% vs 9%), and sensory symptoms (42% vs 9%). Also, their symptoms persisted longer (median duration: 7 vs 4 days). Interestingly, no difference in nasopharyngeal viral load could be detected by age, or even between symptomatic and asymptomatic children [27].

In the first reports, it appeared that transmission from a household member accounted for nearly 75% of infections in children, with an adult member being the first case in the household in two-thirds of family clusters [24]. A further meta-analysis reported that only 3.8% of transmission clusters were identified as having a pediatric index case [28].

A systematic review and meta-analysis of 65 articles representing 1214 children aged 0-4 years with laboratory-confirmed COVID-19 infection showed that about 50% were infants, 53% were male, 43% were asymptomatic and 7% had a severe disease that required intensive-care-unit admission. The study also confirmed vertical transmission, because 3.6% of newborns from COVID-19 infected mothers were COVID-19 positive. Among those 1214 children, one death was recorded [29]. Management using a combination of supportive care, standard practice intensive care management, and anti-inflammatory agents were most likely to lead to recovery, while long-term sequelae of viral exposure remained unknown at this point of the pandemic [30].

The risk for the severe form was the highest in the neonatal age group, among males, children with lower respiratory tract disease, with pre-existing medical conditions and obesity. Vertical transmission from mother to child was confirmed. In mid-2020, the first reports of the very rare multisystem inflammatory syndrome (MIS) appeared in connection with SARS-CoV-2 infection, with fever, involvement of multiple organs and systems and positive laboratory markers of inflammation [20,25]. The percentage of asymptomatic cases vaccinated with BCG was not significantly higher than that without BCG vaccination (30.7% vs 46.2%, *P* = 0.203). Initial symptoms were not related to the immunized influenza vaccine (*P* = 0.267) [31]. Seropositive young adults had about one-fifth the risk of subsequent infection compared with seronegative individuals. Although antibodies induced by initial infection were largely protective, they did not guarantee effective SARS-CoV-2 neutralisation activity or immunity against subsequent infection [32,33].

An interesting question was the duration of humoral immune response in children. Some authors detected specific IgGs 6 months after infection in children who were either asymptomatic or had mild symptoms [34]. However, other studies suggested waning of antibody responses in children 4-6 after SARS-CoV-2 infection, while parents had somewhat higher levels, but still waning [35,36].

Early studies suggested that vertical transmission of SARS-CoV-2 was rare, but it seemed associated with an increased risk of premature birth and higher neonatal morbidity and mortality. Maternal infection should not affect either the mode of delivery or cord clamping routines and rooming-in and breastfeeding were not counter-indicated. Antibodies against SARS-CoV-2 seemed to cross the placenta and could also be found in breast milk, thus providing some protection for the newborn [37]. Like adults, children could also have long-term complications of COVID-19, which may include neurological and cardiac morbidity [38].

Epidemiological studies showed that outbreaks associated with child care facilities, defined as “two or more laboratory-confirmed and epidemiologically linked cases at a facility within 14 days”, occurred in about 6% of the facilities, but accounted for about 50% of total cases [39]. Meanwhile, between March and April 2020, a rapid decline in cases of acute respiratory infections was epidemiologically observed across the USA, especially in the proportion of RSV and influenza, which was attributed to community mitigation measures against SARS-CoV-2 [40]. On the other hand, concerns over the indirect effects of the anti-epidemic measures on child health included psychiatric morbidities, loss of education, unhealthy lifestyle and increased neglect of children [20].

Based on the information from the Coronavirus Disease 2019-Associated Hospitalization Surveillance Network (COVID-NET) in 2021, CDC examined COVID-19-associated hospitalizations among adolescents aged 12-17 years. Among 204 adolescents who were likely hospitalized primarily for COVID-19 from January to March 2021, 31% were admitted to an ICU and 5% required invasive mechanical ventilation, with no deaths occurring. From March to April 2021, weekly adolescent hospitalization rates fluctuated from 2.1 per 100 000 in early January 2021, then 0.6 in mid-March, and then 1.3 in April. Cumulative COVID-19-associated hospitalization rates in the extended period between October 2020 and April 2021 were 2.5-3 times higher than were influenza-associated hospitalization rates in the preceding three influenza seasons (2017-2020), based on the Influenza Hospitalization Surveillance Network (FluSurv-NET) [41,42].

An umbrella review covering published systematic reviews from December 2019 to April 2021 studied epidemiological, clinical and biomarker profiles of pediatric patients infected with COVID-19. A total of 38 systematic reviews covered 1145 studies and 334 398 children and adolescents. The review showed that patients with comorbidity were at higher risk, with 21% (95% CI = 17.8%-24.7%) of the patients being asymptomatic, and that mortality rate was 0.12% (95% CI = 0.036%-0.246%). It confirmed that fever (in 48%-64%) and cough (in 35%-56%) were common symptoms, and ground-glass opacities (in 27%-62%) were the most frequent radiographic observation. Increased C-reactive protein (CRP) level was found in 14%-54%, lactate dehydrogenase (LDH) in 12%-50% and D-dimer in 0%-67% [43].

In low and middle-income countries (LMICs), children and young people contribute to a higher proportion of the total population. Therefore, the features of COVID-19 among CYP are very relevant for society. They are also considerably more likely to suffer from HIV or malnutrition. The pandemic will worsen their situation because it will divert resources from child health to address the needs of the affected adults. Lack of sanitation, running water, universally accessible masks and crowding will likely facilitate the transmission of SARS-CoV-2 in this population. It is expected that this will increase poverty, disrupt schooling, prevent access to school feeding and health facilities, and interrupt other important vaccinations [44].

By the end of 2020, SARS-CoV-2 was identified on antemortem or post-mortem sampling, and eighteen (90%) of 20 deaths with SARS-CoV-2 infection were in children younger than 1 year. Lung histopathologic features included diffuse alveolar damage in 55%, type 2 pneumocyte proliferation in 55% and hyaline membrane formation in 36%, with the culture-confirmed invasive bacterial disease also evident in 55% of COVID-19 attributed deaths. Interestingly, COVID-19 was in the causal pathway of 11% of all childhood deaths under surveillance [45,46].

Studies conducted throughout 2021 brought further understanding to a spectrum of clinical symptoms and patterns of spread of the COVID-19 in children [47-70]. A study in Belgium found anti-SARS-CoV-2 antibodies in 4.4% of children in the low transmission region and 14.4% of children in the high transmission region. A higher risk of seropositivity was associated with a contact with a confirmed case (RR = 2.9), participation in extracurricular activities (RR = 5.6), or a caregiver health care worker who had contact with COVID-19 patients (RR = 2.2). The study showed that, when SARS-CoV2 circulation in the community is high, this will be reflected in the pediatric population with similar infection rates [47]. A clear association was established between the duration of the COVID-19 symptoms in children and the generated IgG units [48].

Big studies in the USA, from the COVID-NET collaboration in 14 US states, calculated that between March 1, 2020, and August 14, 2021, the cumulative incidence of COVID-19-associated hospitalizations was 49.7 per 100 000 children and adolescents. Furthermore, during June 20-July 31, 2021, the hospitalization rate among unvaccinated adolescents (aged 12-17 years) was 10.1 times higher than that among fully vaccinated adolescents [49]. Importantly, the prevalence of severe disease after the Delta variant became predominant (June 20-July 31, 2021) was similar to the same indicator earlier in the pandemic (March 1, 2020-June 19, 2021) [49]. Analyses from the CDC focused on the new COVID-19 cases, emergency department visits and hospital admissions among persons aged 0-17 years between August 1, 2020-August 27, 2021 (the Delta variant period) showed that COVID-19-related emergency department visits and hospital admissions in the states with the lowest vaccination coverage were 3.4 and 3.7 times that in the states with the highest vaccination coverage, respectively. In the hospitals, the proportion of COVID-19 patients aged 0-17 years admitted to an ICU ranged from 10%-25% during August 2020-June 2021 [50]. Another study in the USA involved 45 US children's hospitals with 4063 hospitalizations [57]. The severity of COVID-19 was moderate in 79%, severe in 11%, and very severe in 9%. Factors associated with hospitalization included obesity and diabetes (adjusted OR = 10.4), asthma (OR = 1.4), cardiovascular disease (OR = 5.0), immunocompromised condition (OR = 5.9), pulmonary disease (OR = 5.3), and neurologic disease (OR = 3.2). Greater severity of symptoms was also associated with Black or other non-White race and age greater than 4 years. Further US-based studies confirmed the important role of obesity and a past medical history of diabetes as significantly more prevalent in hospitalized children, while the history of asthma or lower socioeconomic status did not reach statistical significance [66]. Among children aged ≥12 years in Oregon and Washington states in the USA, the incidence of SARS-CoV-2 infection in the July-September 2021 period was 30.1 per 1000 unvaccinated persons and 8.7 per 1000 vaccinated [67].

In Brazil, during the first weeks of the pandemic, the number of COVID-19 hospitalisations increased in older adults but decreased in younger adults. However, from 2021, the number of hospitalisations changed. Hospitalisations of younger adults increased to reach 44.9%, while hospitalisations of older adults decreased to reach 17.3% [51].

In Canada, the Canadian Paediatric Surveillance Program COVID-19 Study Team reported on 264 hospital admissions involving children with SARS-CoV-2 infection, where 57% of admissions were related to COVID-19 and 38% were incidental infections. Infants (37%) and adolescents (30%) represented most cases. Among COVID-19-related hospitalizations, 35% had the critical disease, and 28% of them required respiratory or hemodynamic support, with 39% having at least one underlying comorbidity [53-55]. Another Canadian study included 1632 participants providing samples from 30 day cares, 22 primary schools, and 11 secondary schools. The mean seroprevalence was 5.8%, but it increased from 3.2% in the fall of 2020 to 8.4% in the spring of 2021. Of the 95 children with SARS-CoV-2 antibodies, 82% were not tested or tested negative with reverse transcription-polymerase chain reaction (RT-PCR) testing, and all experienced mild (52%) or no symptoms (48%). The children of parents who self-identified as belonging to a racial and ethnic minority group were about twice as likely to be seropositive [69].

In Switzerland, the Swiss Paediatric Surveillance Unit collected data from children <18 years with confirmed SARS-CoV-2 infection based on the input from all 33 paediatric hospitals. The analysis included 678 children with a median age of 12.2 years, 46.6% female and 15.6% with comorbidities. Eventually, 126 children were hospitalised and 16 required ICU admission. Comorbidities seemed to be the only factor associated with hospital admission in a multivariable regression analysis (OR = 3.2). Hospitalised children more often presented with fever (76.2%) and rash (12.8%). Complications were reported in 28 children, and cardiovascular complications were the most frequent – in 12 children. In total, three deaths were reported [70].

In Australia, a national prospective study was conducted to characterise SARS-CoV-2 infections among children presenting to paediatric hospitals. There were 381 children with COVID-19, 53% were male and 1.3% had multisystem inflammatory syndromes. Nearly 20% of children had comorbidities. All children recovered and most had mild disease [63]. However, in England, the situation was very different: of about 12 million CYP who lived in England in 2020, there were 3105 deaths, and 61 of those who died were positive for SARS-CoV-2. Among them, 25 were due to SARS-CoV-2 infection, giving a mortality rate of two deaths per million children. It is recorded that 22 deaths were due to COVID-19, while 3 were attributed to the pediatric inflammatory multisystem syndrome. In comparison to all CYP deaths, children older than 10 years, Asian and Black ethnic backgrounds and comorbidities were over-represented [64]. In Russia, a rare case of stillbirth due to antenatal asphyxia with aspiration pneumonia was described. The child was positive for SARS-CoV-2 and immunohistochemical investigations showed viral infection and cellular changes in several organs, including the pancreas, brain, spleen, and adrenals [65].

To improve understanding of the patterns of spread, a study in Utah and New York involved surveillance of 1236 participants in 310 households, with 14% aged 0-4 years, 25% aged 5-11 years, 13% aged 12-17 years, and 47% aged 18 years or older. The incidence rate of SARS-CoV-2 infection in Utah was 3.8 per 1000 person-weeks, and in New York 7.7 per 1000 person-weeks, and the rates were similar by age groups of children. However, the asymptomatic fraction was the highest in 0-4 years (52%), then 5-11 years (50%), 12-17 years (45%), and 18+ years (12%). In households with cases of infections, the mean risk of SARS-CoV-2 infection among all other members was 52% (range: 11%-100%). The study showed that children had similar incidence rates of SARS-CoV-2 infection compared with adults, but a larger proportion of infections among children were asymptomatic [58,59,61].

In Japan, researchers tried to estimate the secondary transmission rate at schools using the results of a real-time reverse transcription-polymerase chain reaction (RT-PCR) screening test. They evaluated 1924 students from 52 schools or preschools. The authors estimated the probability of transmission after each contact at school as approximately 0.5% per contact with the infection prevention measures at schools in Japan, which included hand hygiene, physical distancing, wearing masks, and effective ventilation. Assuming that all children are capable of carrying the infection, contact between an infected case and 20-30 students per day at schools would lead to more than 1 secondary case during the ten days of the infectious period [68].

Some studies tried to understand better the immune response in children, as compared to adults. In children, when compared with adults, the antiviral response resolves faster - within a week of symptoms. Monocytes and dendritic cells are less activated, while genes associated with B cell activation appear earlier in children, but these differences do not have major effects on specific antibody responses [56]. A study in Germany showed that about 60% of antibody-positive children showed very high levels of antibodies against N-protein and the S-protein, with 86% developing a sufficient neutralizing activity regardless of age and sex. Approximately 30% of PCR-positive children did not show seroconversion. Symptoms of SARS-CoV-2 infections are unspecific in children, with antibody responses either being very strong in many children or not detectable at all [60]. In the United States, among the 1038 children, researchers found an anti-SARS-CoV-2 antibody positivity rate of 8.5%, but as many as 66% of seropositive children had no symptoms of COVID-19. Seropositivity in children was much higher than that in adults in the same region at a similar time [62].

## VIRAL LOAD IN CHILDREN AND YOUNG PEOPLE

In the early stages of the pandemic, viral load (VL) estimates for children were missing. It initially seemed the secondary attack rate was lower if the infector was a child, but this needed to be confirmed through larger studies [71]. In one of the first attempts, viral load at diagnosis was compared between 53 children and 352 adults with COVID-19 at some point during the first five days after the first symptoms appeared. The study found no significant differences in SARS-CoV-2 RNA loads between children and adults [72]. At the same time, another report could not prove SARS-CoV-2 carriage in children attending daycare centres during the initial weeks of the epidemic in Belgium [73]. Uncertainty over the viral load and secondary attack rate in children prompted views that dramatic public health measures like school closures were not evidence-based, leading to a heated debate over the issue [74-77].

Further study based on 110 children with COVID-19 with a median age of 10 years showed that age, which ranged widely - from 2 weeks to 21 years - did not seem to affect SARS-CoV-2 viral load [78-82]. All children appeared to be most infectious within the first five days of the onset of symptoms, while the severity of the disease did not seem to correlate well with increased viral loads [78]. Also, the severe disease did not correlate with increased viral loads. SARS-CoV-2 sequences detected among children were representative of those in the community, with novel variants seen in the laboratory. It has been shown that both symptomatic and asymptomatic children can carry high quantities of life, replicating SARS-CoV-2, which can create a potential reservoir for SARS-CoV-2 transmission and evolution of genetic variants [82]. Unsurprisingly, studies based on the virological profiling of asymptomatic children suggested that they do have lower viral loads, as well as faster virus clearance, with no significant age-related differences [79,80]. Among the affected children, activated and senescent cells were higher in adults than in children, while regulatory cells were higher in children. In adults, higher immune activation persisted after 6 months of infection, while children maintained high levels of regulatory cells. The studies showed that adults displayed higher immune activation and lower production of anti-SARS-CoV-2 neutralizing antibodies than children, and this difference in immune response was not related to different viral loads [83]. Laboratory analyses based on saliva samples demonstrated the heterogeneity of the SARS-CoV-2 antibody response in children and showed the additional value of saliva antibody detection [84].

More advanced studies resorted to the use of “systems serology” to examine functional phagocyte and complement-activating IgG response to SARS-CoV-2. In children, these were similar to the acute responses generated in adults with mild disease. However, IgA and neutrophil responses were significantly expanded in adults who suffered from more severe forms of the COVID-19. This prompted interest in the serological signature of those rare children who develop MIS-C. They would typically maintain highly inflammatory monocyte-activating SARS-CoV-2 IgG antibodies. Their IgG levels distinguished them from acute disease in children and were more similar to those in convalescent adults. These findings suggested that the interplay of IgG and IgA may be indicative of disease severity and unexpected complications in children [81].

## DIAGNOSIS AND TESTING

Among children and young people, COVID-19 is diagnosed the same way as in adults. A specimen obtained from the upper respiratory tract is tested for nucleic acid amplification (NAAT) using reverse transcriptase viral polymerase chain reaction (RT-PCR). In addition to confirming the presence of viral genetic material, hospitalized patients will typically have leukopenia and increased levels of inflammatory markers. Chest x-ray findings can be very variable. Computed tomography scans of the chest will occasionally show “ground-glass opacities” that are similar to adults, but non-specific findings are also common [85]. Symptoms in children will be milder, including cough, fever, sore throat, myalgia sneezing, and fatigue, but adults will be more severely affected. Lymphopenia may or may not be present and it seems to be less pronounced than in adults [85,86].

It seems that over a third of hospitalized children show redetectable positivity. This is more common among children from family cluster infections, and also those who had relatively higher white blood cell (WBC) count and longer plasma prothrombin time (PT), while age and gender were not associated with this feature [87].

SARS-CoV-2 antigen-specific Fcγ receptor binding accurately distinguishes COVID-19 patients from healthy individuals, suggesting that SARS-CoV-2 infection induces qualitative changes to antibody Fc, enhancing Fcγ receptor engagement. Higher cross-reactive SARS-CoV-2 IgA and IgG are observed in healthy elderly, while healthy children display elevated SARS-CoV-2 IgM. This finding suggests that children have fewer hCoV exposures, resulting in less experienced, but more polyreactive humoral immunity. Age-dependent analysis of COVID-19 patients confirms elevated class-switched antibodies in the elderly, while children have stronger Fc responses which are functionally different [88,89].

## HYPOTHESES ON THE REASONS FOR MILDER CLINICAL PRESENTATION IN CHILDREN AND YOUNG PEOPLE

The striking early observation in the pandemic – ie, that younger age groups have a considerably milder clinical presentation - has sparked interest from several authors [90-96]. It was clear that it could be suggesting something important about the role of the host in the severity of the disease. Hospitalized children’s case fatality rate in the early stage of the pandemic was estimated to be below 1%, compared with 27% across all ages [90]. The first theories suggested that the expression of angiotensin-converting enzyme 2 (ACE2), a receptor for the SARS-CoV-2 spike protein, in upper airways may be very low in children. Then, it can increase with age. It was also noted that higher expression of ACE2 was associated with SARS-CoV-2 genomic RNA positivity in swabs from upper airways in symptomatic children, but it did not correspond as well with viral load [90]. Another theory suggested that children simply do not develop a maladaptive immune response that leads to acute respiratory distress syndrome (ARDS) in older patients [90].

Soon, further ideas followed. Some authors proposed that children have more frequent contact with seasonal coronaviruses than adults, so they may harbour antibodies that provide cross-reactivity with or without co-clearance with other viruses [91]. Another view of the role of ACE2 was that its expression in young people may be increased so that it facilitates SARS-CoV-2 infection in children. However, at the same time, it limits inflammation and reduces the risk of severe disease [91]. There were speculations that recent vaccinations against other viruses may - in some yet unrecognised way - limit the pathogenicity of SARS-CoV-2 in children [93]. The sixth theory, then, suggested that children may have a more diverse memory T-cell repertoire [91].

Further authors speculated that immunosenescence in older people makes them more likely to suffer a more threatening clinical presentation [92], or that a more frequent co-infection with other viruses may provide protection to children [92]. Some authors suggested that lifetime exposure to smoking may play a role [92].

The discussion on this issue then moved from a speculative hypothesis to exploring some measurable indicators that could provide clues. Several authors compared biomarkers in children and adults to study the possible role of the renin-angiotensin system, indicators of the immune response, levels of interleukin (IL)-6, IL-10, myeloperoxidase, P-selectin, intracellular adhesion molecule-1, the role of lymphocytes and the detection of lung infiltrations [93]. It was also speculated that high-titer immunoglobulin-G antibodies against respiratory syncytial virus and mycoplasma pneumonia may carry out cross-protection against SARS-CoV-2 infection in children [93].

Some authors focused on the age-related increase in endothelial damage and changes in clotting function [94]. This further increased interest in measuring density, affinity and distribution of ACE2 receptors, but also transmembrane serine protease 2 [94]. A hypothesis on the role of pre-existing coronavirus antibodies expanded to also study antibody-dependent enhancement and the role of T cells [94]. Theories on the role of immunosenescence further focused on “inflammaging” and a possible role of chronic cytomegalovirus infection [94]. It also became clear that comorbidities, present among older adults, but not children, may be associated with severe COVID-19 in some way. The community interested in the role of vitamin D levels in pathophysiology started looking at its possible contribution [94]. Further hypotheses on why children may be protected included differences in innate and adaptive immunity between children and adults, differences in microbiota, higher levels of melatonin, protective off-target effects of live vaccines, and possibly lower intensity of exposure to SARS-CoV-2. [94].

Interestingly, children with asthma did not appear to be more affected by COVID-19 [95], raising the question of whether atopy may be protective and why, although this issue may not be settled yet. Some theories suggested the possible role of lower IFN-α production, the protective role of eosinophils in the airway, antiviral and immunomodulatory proprieties of inhaled steroids, and allergic sensitization being inversely related to ACE2 expression [95]. The issue is further complicated by the notion that obesity – which is a recognised risk factor in adults – does not seem to associate with increased risk in children with asthma-obesity phenotype [95]. The main concern in children is progression to acute respiratory distress syndrome, which is still sometimes possible – but where “the cytokine storm” that endangers adults is considerably less likely and the mortality is low [96].

## MULTISYSTEM INFLAMMATORY SYNDROME IN CHILDREN (MIS-C)

After establishing that most cases in children and young people are asymptomatic or mild, the research soon focused on the rare exception – the severe form of the disease in children, termed MIS-C [97-111]. It presents initially with fever, feeling very unwell, diarrhoea, shock and it affects two or more organ systems. There may also be the variable presence of rash, conjunctivitis, extremity oedema, and changes of the mucous membrane. In some cases, a progression to multi-organ failure can occur [97,100].

Laboratory tests show elevated markers of inflammation and positivity for SARS-CoV-2 infection. Paediatricians soon realised that MIS-C has similarities with some other conditions in this age group, such as Kawasaki Disease (KD), toxic shock syndrome, and secondary hemophagocytic lymphohistiocytosis/macrophage activation syndrome. The pathogenesis of COVID-19-associated MIS-C likely involves post-infectious immune dysregulation and its treatment requires pediatric intensive care because rapid clinical deterioration can occur among those affected [97,101,106,107,109]. Since the beginning of the pandemic, there was a need to develop a clear case definition and treatment protocol for MIS-C [98,111].

Several groups conducted studies to characterise MIS-C in COVID-19-affected children. Early research found high titers of SARS-CoV-2 RBD IgG antibodies, nucleocapsid protein antibodies and neutralizing antibodies. Children with MIS-C also have detectable RBD immunoglobulin M antibodies, indicating recent infection with SARS-CoV-2. RBD IgG titers correlated well with the erythrocyte sedimentation rate, length of stay in the hospital and ICU [99]. As clinical features initially seem compatible with incomplete KD and Toxic Shock Syndrome (TSS) thought to be related to a cytokine storm, cytokine studies in sera showed increased IL1RA levels (by about 10-20 times), while IL-6 was increased in about half of those affected [104].

Further research managed to assemble a cohort of 539 cases with MIS-C who were then compared with 577 children with COVID-19. MIS-C was more frequent in 6-12 age group. The cases of MIS-C were more likely to show cardiorespiratory involvement and higher neutrophil to lymphocyte ratio, higher C-reactive protein level and lower platelet count. About three in four children with MIS-C were admitted to the ICU, in comparison to less than a half in those with COVID-19. The case-fatality rate among hospitalized children was 1.9% among those who developed MIS-C and 1.4% among those with COVID-19 [105]. Moreover, children with MIS-C can exhibit gastrointestinal symptoms, coagulopathy, shock and an atypical KD syndrome with fever, mucocutaneous lesions, lymphadenopathy and/or cardiovascular events [108].

It is known that certain HLA subtypes are associated with KD. Genetic studies implicated HLA-B*46:01 as the risk allele of severe COVID-19 infection, while blood group O is a protective factor. It is hypothesized that MIS-C could be mediated by a genetic variant of HLA, FcγR, and/or antibody-dependent enhancement (ADE), leading to hyperinflammation associated with T helper 17 (Th17)/Treg imbalance and increased levels of Th17/Th1 mediators - including IL-6, IL-10, IP-10, IFNγ and IL-17A. At the same time, Treg-signalling molecules, FoxP3 and TGF-β are decreased. Intravenous immunoglobulin (IVIG) – alone or combined with corticosteroids - is the standard treatment for KD, KDSS, and MIS-C [108]. Children with MIS-C are significantly more likely to develop pleural effusions on chest radiograph (in 82%) and a lower zone predominance of pulmonary opacifications (in 100%). On abdominal imaging, they have signs of intra-abdominal inflammatory changes [103].

The diagnosis of MIS-C cases will likely become even more challenging after vaccine uptake or natural acquired immunity [110,112]. It is unclear why the disease progresses to severe MIS-C in some children, although they have anti-SARS-CoV-2 antibodies. One explanation could be the presence of the antibody-dependent enhancement (ADE) as a mechanism underlying the clinical syndrome. If true, this should be considered in vaccine development against SARS-CoV-2 [100]. Further studies implied the pathogenesis consistent with the postinfectious inflammatory syndrome, characterised by an elevation in all cytokines and markers of recent T-cell activation (sIL-2R) despite a strong and specific humoral response to SARS-CoV-2 [102,113].

## EARLY RESEARCH ON TREATMENT EFFECTS

Although most SARS-CoV-2 infections in children were either asymptomatic or mild, the emergence of occasional hospitalizations, the presence of particularly vulnerable children in the population and the rare cases of Multisystem Inflammatory Syndrome prompted research into treatments [17,85,114-119]. The presence of pneumonia, severe disease or critical illness would require hospital admission and aggressive management for acute lung injury, shock and/or multiorgan dysfunction. The early opinion from expert panels suggested that early intubation should be preferred over non-invasive ventilation, or overheated, humidified, high flow nasal cannula oxygen in children. This early advice was based on concern that the latter two approaches may generate aerosols, which would increase the risk of infection in hospital staff.

Later, severe COVID-19 in children was defined as a “supplemental oxygen requirement without the need for non-invasive or invasive mechanical ventilation or extracorporeal membrane oxygenation (ECMO)”. Remdesivir for 5 days was the first medication suggested for treating children, but preferably as part of a clinical trial, to assist better understanding of its effectiveness in younger age groups. Remdesivir was also suggested for critically ill children who required either invasive or noninvasive mechanical ventilation or ECMO. Hydroxychloroquine or Lopinavir-Ritonavir, or other protease inhibitors, were not recommended for COVID-19 in children as they didn’t show effectiveness in clinical trials in adults [115]. Early antivirals that were being used among rare children with a particularly severe course of the disease at the beginning of the pandemic included hydroxychloroquine, remdesivir, lopinavir-ritonavir and oseltamivir. Immunomodulation was also attempted using dexamethasone and other corticosteroids, intravenous immunoglobulin, tocilizumab, anakinra and siltuximab [17,119]. Treatments with azithromycin and convalescent plasma were also attempted [114].

Oxygen therapy was typically administered to hypoxic children, with saturation <92%. Similar to adults, oxygenation was maintained through the use of a high flow nasal cannula, CPAP, or ventilatory support, using low tidal volumes (5-6 cc/kg), high positive expiratory pressure, adequate sedation, paralysis, and prone positioning [114]. However, ventilation management interim guidance in the early stage of the pandemic had to rely on evidence-based guidelines in non-COVID ARDS. Decisions needed to be made between noninvasive positive pressure ventilation and high-flow nasal cannula; and also, between high and lower positive end-expiratory pressure strategies related to lung compliance. Management of acute respiratory failure required individualized titration of noninvasive and invasive ventilation, closely considering preserved pulmonary compliance [118].

In November 2020, the US Food and Drug Administration (FDA) provided Emergency Use Authorizations (EUA) for two new virus-neutralizing monoclonal antibody therapies - bamlanivimab and REGN-COV2 (casirivimab plus imdevimab). These drugs were authorised for the treatment of mild to moderate COVID-19 in adolescents and adults in precisely defined high-risk groups, based on recommendations from a panel of experts from 29 North American institutions. They noted that, while there was limited evidence of modest benefit in adults, there was no strong evidence for the safety and efficacy of monoclonal antibody treatment of COVID-19 in children or adolescents. Based on evidence that was available on December 20, 2020, the panel suggested against routine administration of monoclonal antibody therapy (bamlanivimab, or casirivimab and imdevimab) for treatment of COVID-19 in children or adolescents, including those at high risk of progression to hospitalization or severe disease [116]. At that time, it was expected that the approval of vaccines by the FDA under emergency use authorization for children and adolescents will be a better way to protect those most vulnerable [85,117].

## STUDY OF COVID-19 IN PARTICULARLY VULNERABLE CHILDREN

After the symptoms in children were better understood and the treatment options explored for the MIS-C syndrome, the first vaccines to prevent COVID-19 became licensed for adults. This immediately led to an interest in whether particularly vulnerable children could also be vaccinated, and what is their risk of disease and treatment options. A study of nine patients with congenital heart disease showed that two of them died from COVID-19, which led to severe types of congenital heart disease (CHD), worsened arterial blood gases, severe clinical symptoms, a higher mean level of partial thromboplastin time and C-reactive protein [120]. This indicated that any risk of vaccinating children with CHD would likely be much smaller than the risk that COVID-19 posed. A study of 1490 immunocompromised children, defined as those requiring an annual influenza vaccination due to their underlying condition or medication, showed that their shielding worked very well during the pandemic in the UK because no one tested positive for SARS-CooV-2 [121]. In France, the Leukemia Committee of the French Society for the fight against Cancers and Leukemias in Children and Adolescents (SFCE) proposed specific recommendations, in absence of understanding of the risk that COVID-19 poses in these children that would emerge from clinical studies [122]. The paucity of data was also highlighted for pediatric solid organ transplant (SOT) recipients, with uncertainty over both the SARS-CoV-2 infection and the vaccination in terms of safety, immunogenicity, and vaccine efficacy. It was stressed that trials that would include pediatric SOT recipients are much needed and that some are already under way to fill this knowledge gap [123]. In Italy, paediatric oncology units reported on a cohort of 89 adolescent and young adult patients among whom 80 agreed to vaccination. The nine who refused said that they feared side effects, did not believe in the pandemic or did not have trust in the scientific community. Still, the rate of vaccine uptake was higher than in the Italian general population [124]. It was also highlighted that vaccination of children with developmental disabilities should be carefully considered [125].

In a French paediatric oncology institution, a study showed that patients with cancer should be expected to have a severe or critical episode in about 20% of those infected. The study retrospectively evaluated the safety and efficacy of the BNT162b2 mRNA COVID-19 vaccine in adolescents and young adults (AYA) with a solid tumour, aged 16 or over, who were under treatment for a solid tumour or within 6 months after treatment. They received two administrations of the vaccine 3 weeks apart. Of 23 patients with solid tumours who were offered vaccination, nine refused. Vaccines were well tolerated, with five patients having no side effects after the first injection and four with no side effects after the second injection. The main local reactivity symptoms were mild pain at the site of injection and fatigue. Seven of ten patients had positive serology before the second vaccine injection, and nine of ten patients had positive serology one month after the second injection. Furthermore, all patients with seroconversion had positive COVID-19 neutralisation test and no patient developed COVID-19 [126]. These findings suggest a good safety profile and good efficacy of the BNT162B2 vaccine in people aged 16 years or over with solid tumours [126].

The BNT162b2 COVID-19 mRNA vaccine stimulated higher positive antibody responses in adolescents and young adults who were kidney transplant recipients. Importantly, antibody titers after vaccination were significantly lower than after COVID-19 infection, suggesting that a longer time may be required to mount appropriate humoral immunity to vaccination in KTR [127].

Some authors suggested that vaccines should be considered for implementation at child and adolescent psychiatric hospitals [128]. Others critically reviewed pre-and post-marketing evidence on the potential benefits and risks of marketed COVID-19 vaccines in special cohorts that included immunocompromised children, those with predispositions to allergies, children with other comorbidities [129,130], and those with Down syndrome [131]. It was shown that hospitalised minors with Down syndrome had a higher incidence of respiratory symptoms, fever, and several medical complications from COVID-19 than control patients [131]. Significant risk factors for their hospitalisation were older age, obesity, and epilepsy, and they were generally more prone to severe clinical presentation. Mortality rates were low in all paediatric COVID-19 patients, contrasting with previous findings in adults with Down syndrome who had higher mortality [131]. Among parents of children with an autism spectrum disorder in Southern California, approximately 35% intended to vaccinate their child [132]

Vaccine acceptability was also studied among caregivers of childhood cancer survivors. As many as 21% of caregivers expressed hesitancy to vaccinate themselves and 29% expressed hesitancy to vaccinate their children with cancer. Caregivers who expressed confidence in their federal government's response to COVID-19 were six times more likely to express willingness to get vaccinated themselves and three times more likely to vaccinate their child with cancer. This research highlighted the need for COVID-19 vaccination education and outreach [133].

Researchers have also studied antibody response to the BNT162b2 SARS-CoV-2 vaccine in paediatric patients with inflammatory bowel disease treated with anti-TNF therapy. They observed attenuated antibody responses to BNT162b2 in children on the treatment in combination with an immunomodulator, but in most patients antibody responses were still acceptable and comparable with adults. These findings highlighted the importance of booster doses to achieve complete protection in vulnerable patients, prompting future investigations to determine the duration of protection by the immune response [134]. Another similar group of especially vulnerable patients are adolescents with juvenile idiopathic arthritis treated with TNF inhibitors. The research showed that mRNA vaccines develop satisfactory immunogenicity at 1 and 3 months post immunisation. The vaccine assures an adequate humoral response against SARS-CoV-2 [135].

## CONCLUSION

Research to characterise SARS-CoV-2 infection and COVID-19 in children and young people evolved very rapidly during 2020 and 2021. The early research was focused on understanding the symptoms and spreading of the SARS-CoV-2 among pre-school and school-aged minors. Then, information on viral load in children emerged, leading to optimization of diagnosis and testing in this age group. Given that minors had milder symptoms than adults, several hypotheses were proposed to explain this observation, but the debate is still ongoing. In very rare instances, children would develop a MIS-C. Clinical and biochemical features of this condition were gradually documented, and treatment options tested. The effectiveness of treatment using different antiviral, immunomodulatory or supportive therapies, including assisted respiration and ECMO, was examined and treatment guidelines developed. Then, global research efforts focused on protecting particularly vulnerable children, especially after the first vaccines were licensed for the adult population. This gave rise to a complex discussion on an opportunity for vaccinating children and young people to prevent the disease in this age group.
